# Trade-off among different anti-herbivore defence strategies along an altitudinal gradient

**DOI:** 10.1093/aobpla/plw026

**Published:** 2016-07-11

**Authors:** Tomáš Dostálek, Maan Bahadur Rokaya, Petr Maršík, Jan Rezek, Jiří Skuhrovec, Roman Pavela, Zuzana Münzbergová

**Affiliations:** ^1^Institute of Botany, The Czech Academy of Sciences, Zámek 1, Průhonice, CZ-25243, Czech Republic; ^2^Department of Botany, Faculty of Science, Charles University in Prague, Benátská 2, Prague, CZ-12801, Czech Republic; ^3^Department of Biodiversity Research, Global Change Research Institute, The Czech Academy of Sciences, Bělidla 4a, Brno, CZ-60300, Czech Republic; ^4^Institute of Experimental Botany, The Czech Academy of Sciences, Rozvojová 313, Prague, CZ-16502, Czech Republic; ^5^Crop Research Institute, Drnovská 507, Prague, CZ-16106, Czech Republic

**Keywords:** Climate change, defence strategies, elevation, greenhouse experiment, insect herbivory, Lamiaceae, plant–animal interactions, VOCs

## Abstract

The type and intensity of plant–herbivore interactions are likely to be altered under climate change as a consequence of differential dispersal rates of plants and their herbivores. Here, we studied variation in herbivore damage on *Salvia nubicola* in the field and compared its growth and defence strategies against herbivores in controlled conditions using seeds from populations along a broad altitudinal gradient. Our work is one of the first studies to simultaneously measure complex intraspecific variation in plant growth, direct and indirect defences as well as plant tolerance (ability to regrow) as a consequence of herbivore attack simulated by clipping. In the field, we found that plants experienced higher herbivore pressure in lower altitudes. In the greenhouse, plants grown from seeds collected in lower-altitude populations grew better and produced a higher content of phenolic compounds (direct defence) and volatile organic compounds (indirect defence) in response to simulated herbivory. However, there were no differences in tolerance and effect of *S. nubicola* extracts on the model generalist herbivore *Spodoptera littoralis* (direct defence) along the altitudinal gradient. Although we found that *S. nubicola* developed a range of defence strategies, the strategies do not seem to be used simultaneously in all populations even though most of them are correlated with altitudinal gradient. Our finding is in agreement with the current knowledge that co-expression of multiple defences might be costly for a plant, since investment in defensive traits is assumed to reduce the resource availability for growth and reproduction. Our study thus shows the importance of simultaneous study of different defence strategies since understanding these trade-offs could be necessary for detecting the mechanisms by which plants are able to cope with future climate change.

## Abstract (precis)

We found that *Salvia nubicola* distributed along a broad altitudinal gradient developed a range of defence strategies against insect herbivores. The strategies, however, do not seem to be used simultaneously in all populations even though most of them are correlated with the altitudinal gradient along which herbivore pressure is decreasing. Our study thus shows the importance of simultaneous study of different defence strategies since understanding trade-offs among them could be necessary for detecting the mechanisms by which plants are able to cope with changes in plant-herbivore interactions as a consequence of future climate change.

## Introduction

Range shifts to higher latitudes and altitudes are one of the well-documented responses of species to global warming (Grabherr *et al.* 1994; Parmesan 1996; Hoegh-Guldberg *et al.* 2008; Stange and Ayres 2010; Burrows *et al.* 2011; Pateman *et al.* 2012; Pauli *et al.* 2012). Since plants are sessile organisms and cannot disperse upwards as quickly as animals, it is likely that such shifts will lead not only to changes in species distributions but also in the type and intensity of plant–herbivore interactions. Among these, the interactions between plants and insects are likely to have the strongest effects on dynamics of plant populations (Schoonhoven *et al.* 2005; Rasmann *et al.* 2014*c*).

To counter insect aggression, plants have evolved a wide range of defence systems (Rasmann *et al.* 2014*b*). The first type, which includes chemical (such as secondary metabolites) or physical defences that limit plant palatability to insect herbivores, is called direct defences (Schoonhoven *et al.* 2005). The second type is called indirect defences and represents the attraction of natural enemies of the herbivores. Plants are able to produce volatile organic compounds (VOCs) for attraction of organisms at a higher trophic level, such as the predators or parasitoids, that consume or deter the herbivores (Dicke and Baldwin 2010; Lehrman *et al.* 2013). The third type of defence system is tolerance of plants to herbivory which reflects the degree to which a plant can regrow and reproduce after damage from herbivores (Strauss and Agrawal 1999; Schoonhoven *et al.* 2005).

Altitudinal gradients are optimal systems for inferring consequences of shifts in species interactions in varying environmental conditions (Pickett 1989; Körner 2007; Beier *et al.* 2012; Rasmann *et al.* 2014*a*). Compared to much larger-scale latitudinal gradients, altitudinal gradients minimize the confounding effects of historical and biogeographical differences in, for instance, plant and herbivore species pools (Hodkinson 2005). The shifts in abiotic and biotic conditions along altitudinal gradients may promote turnover in plant and insect strategies in communities. Plant defence strategies are, therefore, expected to differ along these gradients (Rasmann and Agrawal 2011; Moles *et al.* 2011; Pellissier *et al.* 2012) and to be affected by climate change (DeLucia *et al.* 2012). According to theory, high-altitude plants, because they experience lower levels of herbivory, are expected to have lower levels of defences against herbivores, compared to their relatives at lower altitudes (Coley and Barone 1996). Alternatively, high-altitude plants usually grow less vigorously because they are limited by nutrient availability in harsh conditions (Körner 2003). Consequently, fast-growing plants are able to withstand higher herbivory since they invest resources to growth instead anti-herbivore defences as predicted by the resource availability hypothesis (Coley *et al.* 1985; Endara and Coley 2011). Nevertheless, little is known regarding how biotic and abiotic factors interact to shape the diversity and amount of plant defensive traits along ecological gradients.

We know a good deal about mechanisms of plant-trait differentiation and local adaptation along steep ecological gradients (Galen *et al.* 1991; reviewed by Núñez-Farfán and Schlichting 2001, Scheepens *et al.* 2013). However, relatively few studies have investigated intraspecific variation in both direct and indirect defence in natural populations of plants (Wason and Hunter 2014; Rasmann *et al.* 2014*c*) and to the best of our knowledge, none have compared direct and indirect defences with tolerance to herbivory and plant growth along environmental gradients. In our study, we investigated variation in anti-herbivore defence and growth at *Salvia nubicola*, Lamiaceae, growing along an altitudinal gradient in the Himalayas. *Salvia* species are known to contain phenolic compounds, which are one of the most important plant defensive substances occurring in plant–herbivore interactions (Daayf *et al.* 2012). They are also one of the dominant groups of secondary metabolites that play a key role in plant responses to other external factors such as radiation, wounding or pathogen attack (Mellway *et al.* 2009), where they can act as anti-feeding agents, antioxidants or signalling molecules (Boeckler *et al.* 2011; Shalaby and Horwitz 2014). While the analysis of secondary metabolites provides information on the content of various chemicals in the plant tissue, it does not provide information on response of real herbivores to these substances. A useful direct approach to see the response of real herbivores is to test the effect of methanolic extracts on feeding, development and mortality of a generalist herbivore (Pavela 2012). *S. nubicola* and most species from Lamiaceae are also well known for their production of range of volatile organic compounds, specifically essential oils stored in their glandular trichomes (Kesselmeier and Staudt 1999; Melkani *et al.* 2010). *S. nubicola* is easy to cultivate and has wide distributional range from 2100 to 3600 m a.s.l. (Press *et al.* 2000). Moreover, it is severely damaged by insect herbivores. All of these factors make *S. nubicola* an ideal model species for assessing different defence strategies along a wide altitudinal range.

In our study, we observed natural herbivory levels along an entire *S. nubicola* altitudinal distribution from 2050 to 3580 m a.s.l. in Manang valley, central Nepal, Himalayas. Since localities along the altitudinal gradient may differ in both biotic and abiotic environmental factors (vegetation composition, soil composition, slope, aspect, etc.) which might also influence herbivory and plant growth, the plants needed to be studied in standard conditions so that we could assess genetically based differences in defence strategies among plants from different populations. We thus performed a greenhouse experiment with plants grown from seeds from populations along the altitudinal distribution. We measured plant growth and all three types of defence strategies, i.e. direct chemical defences (content of phenolic compounds in the leaves and effects of methanolic extracts on a model generalist herbivore), indirect defences (the production of volatile organic compounds – VOCs) and tolerance (the ability of plants to regrow after herbivory). Specifically, we asked the following questions: (i) Is the rate of herbivore attack affected by altitude in natural conditions? (ii) Are there any differences in growth, tolerance and direct and indirect defences to herbivory among populations in standard conditions? (iii) Can these differences be explained by altitude of plant origin? (iv) Is there any correlation between plant growth, tolerance and direct and indirect defences to herbivory?

We hypothesized that herbivore damage in natural populations will decrease with increasing altitude. Although differences among populations are also driven by biotic and abiotic environmental factors, we expect altitude to be the main driver. Plants from lower altitudes will be more adapted to herbivore damage and thus display higher levels of tolerance to herbivore damage as well as direct and indirect defences. Since investment to multiple defensive traits is assumed to reduce the resource availability for growth, trade-off between growth and different types of defences is expected.

## Methods

### Study species

*Salvia nubicola* is a perennial herb with quadrangular stem growing up to 60–150 cm. Leaves are stalked, dentate and hairy with hastate base. Flowers are yellow in large spreading panicles. Flowering and fruiting occurs from June to September. The species is distributed in western and central Nepal from 2100 to 3600 m a.s.l. in open humus rich grounds. Outside Nepal, it can be found also in Afghanistan, Pakistan, northern India, Bhutan and Tibet (Press *et al.* 2000). The plants are avoided by large herbivores but are severely damaged by insects (Rokaya *et al.*, unpublished data).

The plants are used by humans as food and medicine. Seeds are roasted and used as condiment. The decoction of the root is given in cases of fever (Manandhar 2002). The young petioles are chewed raw or spiced and used as condiment.

### Study area

The study area was chosen along the altitudinal gradient from 2050 to 3580 m a.s.l. at 27 different populations of *S. nubicola* in the Manang district (Table 1). Population sites were selected to represent altitudinal gradient through the valley with frequent *S. nubicola* occurrence. We selected all populations on the gradient that were at least 500 m far apart and consisted of at least 50 flowering plants. Manang is a part of Annapurna Conservation Area in central Nepal, extends from east to west and is situated between 28°37′56″–28°39′55″ N latitude and 83°59′83″–84°07′97″ E longitude. There are strong differences in temperatures on the altitudinal gradient—mean month air temperature lapse rate of 1.9 °C m in July and 4.7 °C m in January (Rokaya *et al.* unpublished data). The climate is dry, characteristic to the trans-Himalayan region. Due to the rain shadow of the Annapurna massif, the mean annual precipitation is around 400 mm (ICIMOD 1995). Soil moisture decreases from east to west in the valley, and the south facing slopes are significantly drier than those facing north (Bhattarai *et al.* 2004). Vegetation is dominated by *Pinus wallichiana*, which is abundant on the north aspect from the lower belt up to 3500 m asl. It is replaced by *Abies spectabilis* and *Betula utilis* in higher altitudes. *Juniperus indica* and *Rosa sericea* are the dominants in the dry south facing slopes (Miehe 1982). The ground layer consists of scattered patches of different herbs.

**Table 1.** List of 27 studied *S. nubicola* populations in Annapurna Conservation Area, Nepal with their altitude and geographical position (WGS 84). ‘+’ in columns field herbivory, growth, tolerance, phenolic compounds, methanolic extracts and VOCs indicate that the given characteristics was studied in that population.

Altitude	Latitude	Longitude	Field herbivory	Growth	Tolerance	Phenolic compounds	Methanolic extracts	VOCs

2050	28°31.913′	84°20.851′	+	+	+			
2175	28°32.019′	84°19.926′	+	+	+			
2275	28°31.746′	84°19.135′	+	+	+	+	+	+
2619	28°31.448′	84°18.442′	+	+	+			
2630	28°33.024′	84°16.040′	+	+	+	+		
2664	28°31.573′	84°18.208′	+	+	+	+	+	+
2677	28°31.790′	84°18.162′	+	+	+			+
2695	28°33.289′	84°14.065′	+	+	+	+		+
2700	28°33.470′	84°15.228′	+	+	+			
2716	28°32.608′	84°17.472′	+	+	+			
2729	28°33.859′	84°12.784′	+	+	+	+	+	+
2826	28°33.478′	84°13.439′	+	+	+			
2908	28°34.333′	84°11.634′	+	+	+			
3000	28°34.857′	84°11.030′	+	+	+			
3145	28°35.446′	84°11.110′	+					
3177	28°36.152′	84°10.296′	+	+	+	+		+
3213	28°37.018′	84°08.803′	+					
3214	28°36.376′	84°09.745′	+	+	+	+	+	
3220	28°36.648′	84°09.644′	+	+	+			
3222	28°36.505′	84°10.043′	+	+	+	+		+
3223	28°36.713′	84°09.379′	+	+	+			+
3255	28°37.201′	84°08.259′	+	+	+			
3262	28°37.379′	84°08.102′	+	+	+		+	
3356	28°37.686′	84°07.196′	+					
3487	28°39.565′	84°02.208′	+					
3493	28°39.634′	84°01.882′	+					
3580	28°40.175′	84°01.053′	+					

### Field herbivory in natural populations

To assess variation in herbivore damage on *S. nubicola* plants along the altitudinal gradient, 20 mature flowering plants were selected at the time of flowering (July 2014) at each of the 27 localities (Table 1). We randomly selected plants with at least two flowering stems and at least 1 m apart. At each plant individual, herbivore damages of five different leaves along the stem were noted in percentages and the values were averaged. All damage found in the leaves of *S. nubicola* was holes caused by leaf chewers. We observed caterpillars from Noctuidae family feeding on *S. nubicola* and assume they are the main herbivores.

### Greenhouse experiment

We collected seeds of *S. nubicola* in 21 out of the above mentioned 27 populations along the altitudinal gradient from 2050 to 3262 m a.s.l. (Table 1). Seeds were collected from five adult plants randomly selected within each population during seed maturation in October 2013. Seeds from six populations were not available because of earlier seed maturing time in some populations.

At the end of February 2014, 15 fully developed seeds from each of five adult plants from 21 populations were sown in Petri dishes and germinated in the growth chamber (12 h 10 °C night, 12 h 25 °C day). Germination was checked at 7-day intervals and seedlings were transplanted to the seedling tray and kept in the same growth chamber until the end of April 2014.

*Plant growth***.** At the beginning of May 2014, 20 randomly chosen seedlings from each population were transplanted to 2 L pots filled with mixture of steam sterilized common garden soil and sand (1:1) and transferred to the greenhouse. To protect plants from freezing, the temperature in the greenhouse was not allowed to drop below 5 °C. Every month between May and September 2014, we measured the number of stems, number of fully developed leaves on the longest stem and length of the longest stem. The planned design of this study was 21 populations × 20 plants = 420 plants. Due to lower germination rate or seedling mortality in some populations, total plant number in the experiment was 374 (always at least 12 plants per population except for population from altitude 3255 m a.s.l. from which only six plants were available).

*Tolerance to herbivory*. To assess variation in plant tolerance to herbivore damage, we used a widely employed generalist herbivore known to feed on plants from over 40 families worldwide, the Egyptian cotton leafworm *Spodoptera littoralis* (Brown and Dewhurst 1975; Ali and Agrawal 2012). Its extreme polyphagy makes it an excellent bioassay species for experiments with leaf palatability (Kempel *et al.* 2011) although it is not native to our system. Plants from the Lamiaceae family were shown to contain compounds that inhibit growth and survival of *S. littoralis* (Pavela 2012; Pavela *et al.* 2014). The test caterpillars originated from a laboratory stock (Laboratory of quarantine organisms, Department of Entomology, Crop Research Institute in Prague, CZ) bred on artificial Stonefly Heliothis diet (Wards Natural Science Inc, USA) and thus were not adapted to our study species. Larvae of *S. littoralis* were all of identical age (born within the same day) and were 21 days old when the experiment was initiated. The breeding was carried out in the laboratory at 22 ± 1 °C, relative humidity 40–60% and a 16:8 (L:D) photoperiod in plastic boxes. In the middle of July 2014, one-third instar larva was placed on the petiole of the lowest leaf on half (i.e. 10) of the plants from each population. Each plant was covered with fine mesh net to prevent larvae from escaping or moving to another plant. The other half of the plants was covered with net without larvae addition as a control treatment. After 14 days, no living larvae of *S. littoralis* were present and there was no or very low herbivore damage on each plant (maximum up to several cm^2^ in very few cases). The result was the same when we repeated the experiment with addition of three *S. littoralis* larvae directly on the leaves. High larvae mortality could be caused by production of specific compounds toxic for *S. littoralis* or by plant physical traits such as leaf hairs limiting their feeding as shown in other studies (Kamel 1965).

**Figure 1.**
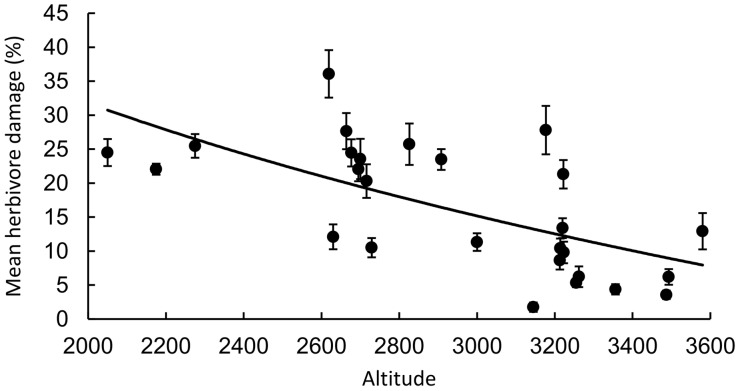
Relationship between altitude and herbivore damage on S. nubicola recorded in the field. Population means and SE are shown.

We thus decided to simulate herbivore damage by mechanical clipping. In the middle of August 2014, we cut off half of the leaf area at every second leaf with scissors on plants with previous *S. littoralis* addition. At each side of the leaf, four large triangles were cut off the leaf without affecting the middle vein. The level of defoliation after clipping treatment was thus 25% which is within the range of observed intensity of herbivory in the field (Fig. 1).

Clipped and unclipped plants were kept in two separate parts of the greenhouse to prevent them from affecting neighbouring plants and to avoid mixing VOCs from clipped and unclipped plants (details on VOCs below). Both greenhouse chambers had the same conditions (temperature, light and watering conditions). Our original plan was to have four plants grown from seeds collected from each of five mother plants per each population along the altitudinal gradient and to treat two of them with clipping and take two of them as a control. However, because of the variability in seed germination and seedling survival this was not possible and we thus did not include information on mother plant identity in further analyses.

In September 2014 (4 weeks after clipping), all plants were measured, harvested, shoot and root biomass was separated, dried at 40 °C for 72 h and weighed. The parts of the leaves clipped off during the herbivore treatment in the middle of August 2014 were also dried at 40 °C for 72 h, weighed and their weight was added to shoot biomass and total biomass weight.

*Direct defences I—phenolic compounds.* In our experiment, we focused on changes in concentrations of phenolic compounds related to plant defence including coumarins, lignans, rosmarinic acid and salicin. Coumarins are widely distributed secondary metabolites in plants including the Lamiaceae family and are involved in defence against biotic and abiotic factors (Bourgaud *et al.* 2006). Lignans are phenylpropanoids occurring universally in plants, and that play a significant role in the defence of plants against insects. They act largely as regulators of insect feeding (Harmatha and Dinan 2003). Salicin and rosmarinic acid are well known defence phenolic secondary metabolites against herbivory and infections and are known to cause biological activity of several species including the genus *Salvia* (Petersen *et al.* 2009; Boeckler *et al.* 2011).

In August 2014, we sampled one leaf of *S. nubicola* from the third fully developed leaf floor from fully grown non-flowering plants in the greenhouse experiment described above. We sampled leaves from 5 plants × 8 populations × 2 clipping treatments, i.e. 80 samples. The eight populations were selected to represent the altitudinal gradient and include plants in similar phenological phase (Table 1). Leaves from plants treated with clipping were cut off 48 h after clipping. Sampled leaves were deep frozen in −80 °C until extraction in June 2015 when they were applied to LC/MS/MS (refer File 1 in **SupportingInformation** for more details). We detected measurable content for two coumarins (esculin, esculetin), rosmarinic acid and salicin. One leaf from the third fully developed leaf floor was also cut off from plants from remaining plants and populations where phenolic compounds were not analysed and its dry weight was added to shoot biomass and total biomass weight in the end of the experiment. Leaves sampled for the analyses were also weighed after they had been cut off and their dry weight was recalculated and added to shoot and total biomass of corresponding plants.

*Direct defences II—effects of methanolic extracts on larvae of S. littoralis****.*** When we recorded 100% mortality of larvae of *S. littoralis* in the experiment described in the above section *Tolerance to herbivory*, we conducted an additional experiment in the laboratory to determine the extent of *S. nubicola* toxicity for *S. littoralis*. We thus tested the effects of methanolic extracts from five populations of *S. nubicola* (Table 1) on feeding, development and mortality of *S. littoralis* larvae. The five populations were chosen to represent the altitudinal gradient. Methanolic extracts were extracted from dry *S. nubicola* biomass harvested in September 2014 in the greenhouse experiment. Extract was prepared from biomass from all (usually 10) control plants (not treated with clipping) from each of five populations. First, we assessed lethal doses causing 50% and 90% (LD_50_ and LD_90_) of antifeedancy and feeding deterrence index (FDI) according to the methods described in Pavela *et al.* (2008) and compared the values between the five populations along altitudinal gradient. Second, we assessed particular mechanisms of toxicity and growth inhibition. The effect of methanolic extract of *S. nubicola* plants incorporated into larval diet on food consumption and utilization by *S. littoralis* larvae was thus tested as described in Pavela *et al.* (2008) with following modifications. The extracts were presented in an artificial diet for 10 days. Larvae reared on a control diet were weighed after the second molt (<24 h), and placed individually in Petri dishes (50 mm diameter). The third instar larvae were fed controlled amounts of food containing 0 (control), 0.5, 1.0, 1.6 and 2.5 mg g^−^^1^ of the extract (*n* = 30 for each concentration) and were fed 5 days, which is a period a little shorter than instar duration. At the end of the experiment, the larvae, their faeces and uneaten food were dried and weighed. Fresh weights of faeces and consumed food were determined from dry weight *vs.* fresh weight curves. The nutritional indices: relative consumption rate (RCR), relative growth rate (RGR), efficiency of conversion of ingested food (ECI), efficiency of conversion of digested food (ECD) and approximate digestibility (AD) were calculated as follows:

RCR = I/BaT; RGR = B/BaT; ECI = (B/I) × 100; ECD = [B/I − F)] × 100; and AD = [(I − F)/I] × 100, where I = weight of food consumed; Ba = arithmetic mean of insect weight during the experiment; T = feeding period (d); B = change in body weight; F= weight of faeces during the feeding period (Waldbauer 1968; Farrar *et al.* 1989).

*Indirect defences—volatile organic compounds (VOCs).* To characterize the VOC emissions of *S. nubicola*, we collected VOCs from *S. nubicola* plants in the greenhouse experiment described above in the middle of August 2014. Each plant (including its pot) was transferred to the glass box (35 × 35 × 105 cm) just after clipping described above, left there for 20 min and VOCs were collected for subsequent 20 min from the top of the glass box. We sampled plants from eight populations along the altitudinal gradient × 3 plant replicates × 2 treatments (clipped and unclipped), i.e. 48 plants altogether. Even though using actual herbivores is more realistic, the clipping approach provides a more standardized induction due to possible variation in herbivore damage among populations from different altitudes along the gradient. Due to the limit of samples which could be taken for VOCs per one day, samples were collected during 5 days within a 10-day interval. We collected VOCs emissions from clipped and unclipped plants originating from multiple sites each day so that ‘collection day’ was not confounded with altitude.

Volatile organic compounds (VOCs) were trapped in Tenax TA sorbent tubes (Gerstel GmbH & Co. KG, Mülheim an der Ruhr, Germany) from 2 L of air under the flow of 100 mL/min using Casella Cel Apex Air Sampling Pump equipped with Low Flow Adaptor (Casella CEL, Stokenchurch, UK). Analysis was performed on LECO Pegasus 4D GC × GC-TOFMS system (Leco Corporation, St. Joseph, MI, USA) containing Agilent 7890 gas chromatograph (Agilent technologies, Santa Clara, CF, USA)—see File 2 in **SupportingInformation** for more details.

### Data analyses

The relationship between altitude and mean field herbivory per plant was tested using linear regression. We added 1 to all field herbivore damage data and log transformed the data to meet test assumptions.

In all further described tests, we tested the effect of both population and altitude. Since each population has unique altitude, the effect of each of them was tested separately. This is further described as test of effect of population/altitude.

To describe determinants of plant growth, we tested the effect of population/altitude on number of stems, number of leaves on the longest stem and length of the longest stem of the plants each month from May to September 2014. As the results for plant growth were largely similar for the time periods before and after clipping, only data before clipping (at 3 months growth in July 2014) and data from unclipped plants at the end of the experiment (at 5 months growth in September 2014) are presented further. We also tested the effect of population/altitude on root, shoot and total biomass weight and root:shoot ratio.

To test plant tolerance to herbivory (simulated by clipping), we performed several independent tests using Generalized Linear Models (GLMs) with plant traits (number of stems, number of leaves on the longest stem, length of the longest stem, root, shoot and total biomass weight and root:shoot ratio) as dependent variables and population/altitude and clipping treatment and their interaction as independent variables. Plant size before clipping treatment (July 2014) was taken as a covariate in the tests of tolerance to herbivory. Significant interaction of population/altitude and clipping treatment would indicate different response of plants from different populations/altitudes, i.e. different tolerance to herbivory. The number of stems was tested using Poisson distribution, number of leaves on the longest stem, length of the longest stem, root weight, root:shoot ratio, shoot weight and total biomass weight using Gaussian distribution.

We tested the effect of clipping treatment, population/altitude and their interaction on content of the four detected phenolic compounds (salicin, rosmarinic acid, esculin and esculetin) using ANOVA. Salicin and rosmarinic acid content was log transformed and esculetin content was square root transformed to meet test assumptions.

The lethal doses (LD_50_, LD_90_) and feeding deterrence index (FDI) and their 95% confidence intervals (CI_95_) for third instar *S. littoralis* larvae were calculated for methanolic extracts from *S. nubicola* plants from populations along altitudinal gradient using the Spearman-Karber method with Abbot correction (Hamilton *et al.* 1977). Differences in effect of five concentrations of methanolic extract on relative consumption rate (RCR), relative growth rate (RGR), efficiency of conversion of ingested food (ECI) and approximate digestibility (AD) were evaluated by ANOVA and Tukey’s honest significant difference (HSD) test.

The production of VOCs (peak areas in the chromatograms) was compared between clipped and unclipped plants and among populations/along the altitudinal gradient using multivariate redundancy analyses (Šmilauer and Lepš 2014). Peak areas of 1177 compounds identified in gas chromatography were used as dependent variables and clipping treatment, population/altitude and interactions between population/altitude and clipping treatment as independent variables. Since we detected significant differences in VOC production among four days of VOC sampling, the date of sampling was taken as a covariate in all analyses. We found no significant effect of plant height, number of leaves or proxy of plant biomass (estimated as multiplication of height of the longest stem and number of leaves on the longest stem) on VOC production; thus none of them was used as a covariate in the final tests. In the test of effect of population/altitude, we used clipping treatment as a covariate and vice versa. Data were standardized by both samples and VOCs. Compounds with less than five occurrences in the data were excluded from the analyses as recommended by Šmilauer and Lepš (2014). Because we found no significant effect of population or altitude but significant effect of clipping treatment on VOC production, we selected 30 VOCs that were most strongly influenced by clipping treatment (according to their position on the first axis in the RDA analyses) and tested the effect of population/altitude on each of them separately. Effect of population/altitude on VOC production was performed using GLMs with Gaussian distribution. Only clipped plants were included in the GLMs because VOCs production of unclipped plants was often either very low or could not be detected.

For each plant trait significantly correlated with altitude, we calculated predicted values of plant trait from the lowest altitude (2050 m a.s.l.) to the highest altitude (3580 m a.s.l.). To express the effect of altitude on plant traits, we calculated change in plant trait predicted by model per 100 m of altitudinal change.

To visualize relationships among these traits, we also performed multivariate principal component analyses (PCA) (Šmilauer and Lepš 2014). We used only plant traits significantly correlated with altitude (*P* < 0.1). Altitude and field herbivory were added to the analysis as supplementary variables to show how plant growth and defence traits are related with these variables. Additionally, we constructed a correlation matrix using these plant defence traits. Since all traits were not recorded at every plant and in all populations, we correlated population means. In multivariate analyses, we thus used population means of only six populations where all plant traits were recorded (2275, 2664, 2695, 2729, 3177 and 3222 m a.s.l.). In correlation matrix, we correlated all population means where given pairwise comparison was available (*N* = 6–21).

All the analyses were performed in the R statistical computing program (R Development Core Team 2015) except for multivariate analyses performed in Canoco 5.01 (Šmilauer and Lepš 2014) and correlation matrix created in Statistica 12.6 (StatSoft, Inc. 2015).

## Results

### Field herbivory in natural populations

In the field study during July 2014, we recorded significantly more herbivore damage in plants from populations in lower altitudes compared to plants from populations in higher altitudes (*R*^2 ^=^ ^0.27, *F*_1,537 _=_ _200.36, *P* < 0.001, Fig. 1). There was about 2% less herbivore damage with every 100 m of altitudinal increase ranging from 35% at the lowest population (2050 m a.s.l.) to 5% at the highest population (3580 m a.s.l.).

### Plant growth

Plant growth significantly varied among source populations in the greenhouse experiment. After 3 months of growing in the greenhouse, population origin was responsible for substantial variability in number of stems, number of leaves and length of the longest stem (*R*^2 ^=^ ^0.29, *R*^2 ^=^ ^0.18 and *R*^2 ^=^ ^0.17, respectively, *P* < 0.001 in all cases; see **SupportingInformation** for more details of the results – File 3). Plants from higher altitudes produced more stems, fewer leaves and shorter stems after three months growing in the greenhouse (*R*^2 ^=^ ^0.12, *R*^2 ^=^ ^0.03 and *R*^2 ^=^ ^0.03, respectively, *P* < 0.001 in all cases; Table 2, see **SupportingInformation** for more details of results – File 1). They produced 0.13 more stems, 0.09 fewer leaves and were 0.49 cm shorter per each 100 m of altitudinal increase. After 5 months of growing in the greenhouse, i.e. at the end of the experiment, population origin still explained a significant amount of variability in number of stems, length of the longest stem, shoot, root and total biomass weight and root:shoot ratio at not clipped plants (*R*^2 ^=^ ^0.18–0.24, *P* < 0.03, see **SupportingInformation** for more details of the results – File 3). We found that plants from higher altitudes were shorter and produced less shoot and total biomass and had higher root:shoot ratio (*R*^2 ^=^ ^0.08, *R*^2 ^=^ ^0.06, *R*^2 ^=^ ^0.03 and *R*^2 ^=^ ^0.05, respectively, *P* < 0.012 in all cases; Table 2, see **SupportingInformation** for more details of the results – File 3). They were 0.99 cm shorter and produced 0.18 g less shoot and 0.17 g total biomass and had 0.01 higher root:shoot ratio per each 100 m of altitudinal increase.

**Table 2.** Summed results of effects of clipping, population origin (population) and altitude of population origin (altitude) on traits related to plant growth, tolerance to herbivory, production of phenolic compounds, effect of methanolic extracts and production of volatile organic compounds (VOCs). No interactions between clipping and population/altitude were significant (*P* > 0.05 in all cases) and the interactions are thus not shown (see File 3 in **Supporting Information** for details).

Effect on		Effect of		
		Clipping	Population	Altitude

*Growth*				
Growth: 3 months	No stems	NT	***	+++
	No leaves on the longest stem	NT	***	---
	Length of the longest stem	NT	***	---
*Tolerance*				
	No leaves on the longest stem	+++	n.s.	n.s.
	Length of the longest stem	+++	***	---
	Shoot biomass	--	*	--
	Root biomass	--	.	n.s.
	Root:shoot ratio	n.s.	***	+++
	Total biomass	n.s.	n.s.	n.s.
*Direct defences*				
Phenolic compounds	Salicin	n.s.	***	+++
	Rosmarinic acid	n.s.	*	–
	Esculin	n.s.	n.s.	n.s.
	Esculetin	n.s.	.	n.s.
Methanolic extracts	LD_50_	NT	n.s.	n.s.
	LD_90_	NT	n.s.	n.s.
	FDI_50_	NT	n.s.	n.s.
	FDI_90_	NT	n.s.	n.s.
*Indirect defences*				
VOCs	Total VOCs production	+++	n.s.	n.s.
	(Z)-3-Hexen-1-yl acetate	+++	n.s.	–
	(Z)-Linalool oxide (furanoid)	+++	.	n.s.
	1-Octen-3-yl acetate	+++	.	n.s.
	(Z)-3-Hexenyl isovalerate	+++	n.s.	.-
	Caryophyllene (E)	+++	n.s.	.-
	Valencene	+++	.	n.s.
	δ-Cadinene	+++	n.s.	–

*.P* < 0.1.

* *P* < 0.05.

** *P* < 0.01.

*** *P* < 0.001, n.s. non-significant, NT = not tested.

LD_50_, LD_90_, FDI_50_ and FDI_90_ are the lethal doses (50 and 90%) and feeding deterrence index (50 and 90%) for third instar *S. littoralis* larvae.

‘+’ indicates positive effect of clipping/higher altitude (*P* < 0.05), i.e. clipped plants/plants from higher altitudes produce more stems, ‘ ++’ indicates positive effect of clipping/higher altitude (*P* < 0.01), ‘ +++’ indicates positive effect of clipping/higher altitude (*P* < 0.001).

‘-’ indicates negative effect of clipping/higher altitude (*P* < 0.05), i.e. clipped plants/plants from higher altitudes produce fewer stems, ‘--’ indicates negative effect of clipping/higher altitude (*P* < 0.01), ‘---’ indicates negative effect of clipping/higher altitude (*P* < 0.001).

### Tolerance to herbivory

**Figure 2.**
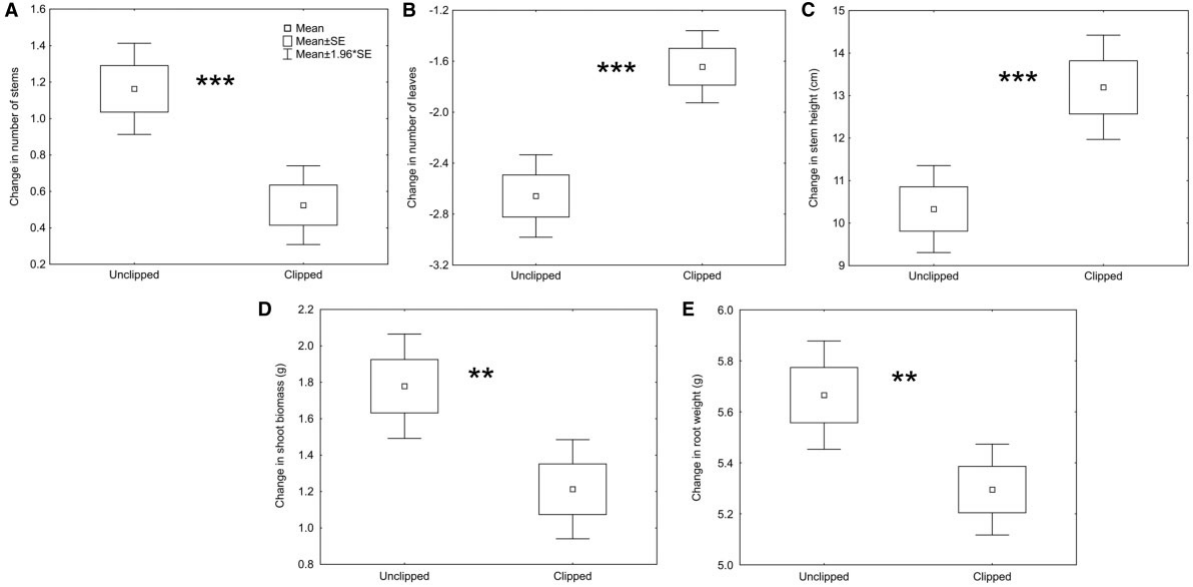
Effect of clipping treatment on change in plant traits after simulated herbivore damage. Effect on change in (A) number of stems, (B) number of leaves, (C) stem height, (D) shoot biomass and (E) root biomass is shown. Asterisks indicate significant differences between not clipped and clipped plants (**P < 0.01, ***P < 0.001). Details of the test results are provided in Supporting Information – File 1.

**Figure 3.**
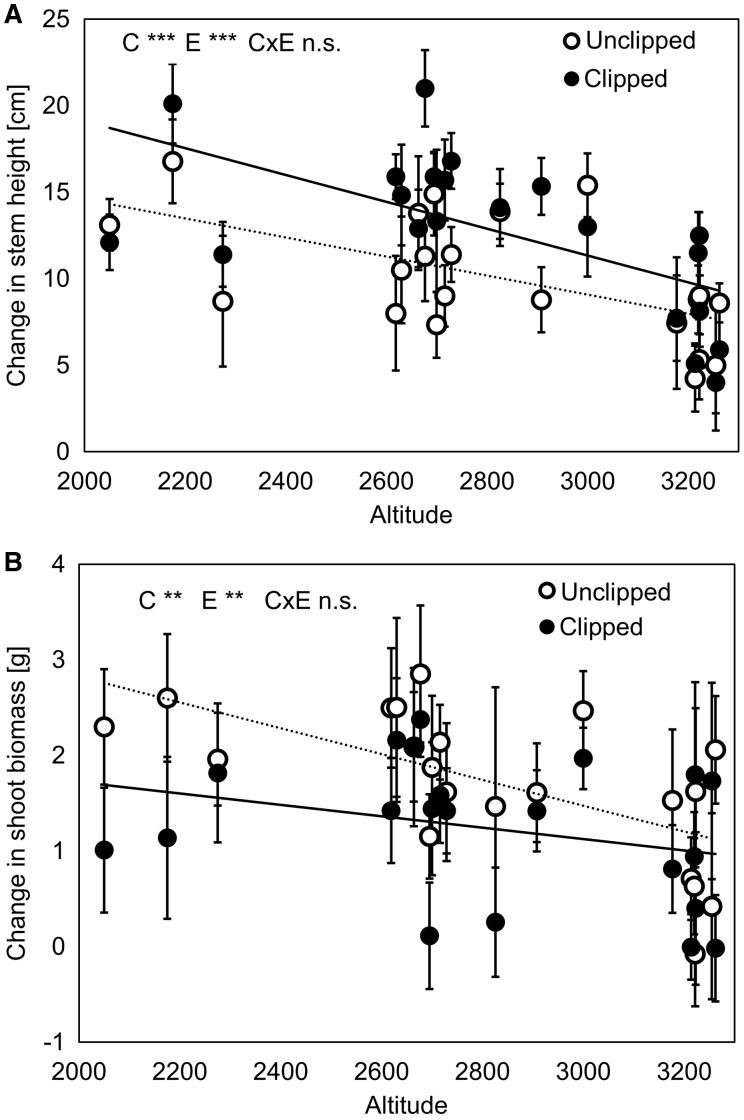
Effect of clipping (simulated herbivore treatment) on plants originating from populations along altitudinal gradient. Effect on change in (A) stem height and (B) shoot biomass is shown. C, E and C × E indicate effects of clipping treatment, altitude and their interaction, respectively. **P < 0.01; ***P < 0.001; n.s. non-significant. Population means and SE are shown. Dotted and solid lines represent data from unclipped and clipped treatment, respectively.

Clipping treatment affected plant traits as well (Table 2, see **SupportingInformation** for more details of the results – File 3). Clipped plants produced 56% fewer stems, 38% more leaves and 27% higher stems than unclipped plants (*P* < 0.001 in all cases; Fig. 2A–C). They also produced 68% less shoot and 7% less root biomass in comparison with unclipped plants (*P* = 0.002 and *P* = 0.006, respectively; Fig. 2D and E). Plant populations produced higher stems, more shoot biomass and had lower root:shoot ratio when they originated from lower altitudes compared to higher altitudes (*R*^2 ^=^ ^0.03, *P* < 0.001; *R*^2 ^=^ ^0.01, *P* = 0.003; *R*^2 ^=^ ^0.03, *P* < 0.001; respectively; Fig. 3, see **SupportingInformation** for more details of the results – File 3). In contrast to our expectation, there were no significant interactions between population/altitude and clipping treatment. This indicates that the effect of altitude was comparable for clipped and unclipped plants and plant response to clipping was very similar across populations/altitudinal gradient (Fig. 3, see **SupportingInformation** for more details of results – File 3).

### Direct defences I—phenolic compounds

**Figure 4.**
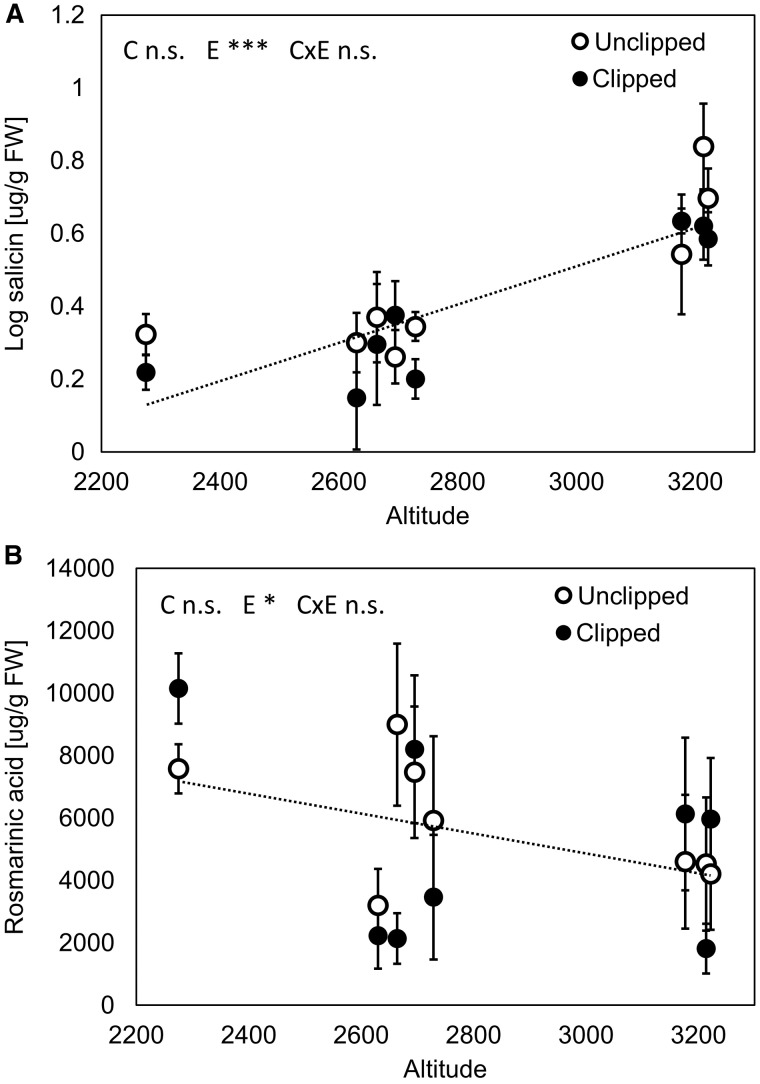
Effect of clipping (simulated herbivore treatment) on production of secondary metabolites—(A) salicin and (B) rosmarinic acid. C, E and C × E indicate effects of clipping treatment, altitude and their interaction, respectively. **P < 0.01; ***P < 0.001; n.s. non-significant. Population means and SE are shown. Dotted line represent data from both unclipped and clipped treatment because no effect was clipping treatment was found.

While we recorded no effect of clipping treatment on production of any of the studied phenolic compounds (*P* > 0.183 in all cases, df = 74), we found a significant relationship between altitude and production of salicin and rosmarinic acid. Plants from higher altitudes produced three times more salicin (*R*^2 ^=^ ^0.37, *F*_1,75 _=_ _43.22, *P* < 0.001; Fig. 4A), in contrast to the hypothesis that plants from high altitudes should produce less defence compounds. In concordance with the hypothesis, there was about two times less rosmarinic acid in plants from higher compared to lower altitudes (*R*^2 ^=^ ^0.06, *F*_1,75 _=_ _4.72, *P* = 0.03; Fig. 4B). Plants produced 0.35 µg/g FW more salicin and 337 µg/g FW less rosmarinic acid per each 100 m of altitudinal increase.

### Direct defences II—effect of methanolic extracts on *S.* *littoralis*

**Figure 5.**
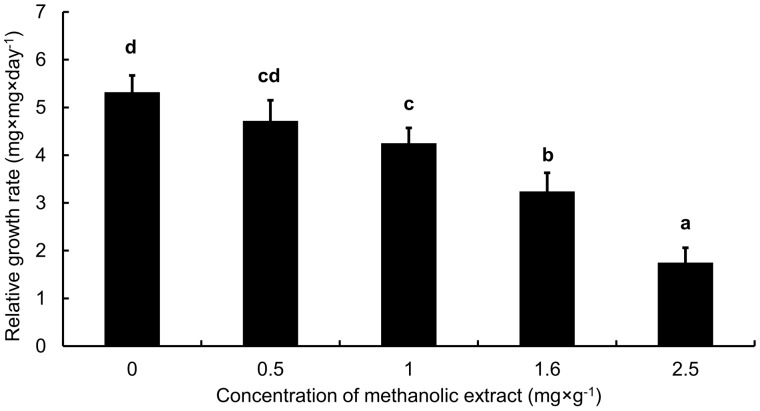
Effect of methanolic extracts from S. nubicola on relative growth rate of *Spodoptera littoralis* larvae. Methanolic extracts were extracted from plants from population at 2729 m a.s.l. Columns sharing the same letter are not significantly different (*P* > 0.05). Means and SE are shown.

In contrast to other defence strategies, the effects of methanolic extracts were not tested in response to clipping treatment but we assessed how extracts from undamaged plants from different populations affected a generalist herbivore. The methanolic extracts from five populations of *S. nubicola* caused slower feeding and development and higher mortality of *S. littoralis* larvae. However, there were no differences among populations or along altitudinal gradient (95% confidence intervals were largely overlapping; see **SupportingInformation** for more details of the results – File 4). Since there were no differences in feeding, development and mortality effects, detailed analyses of particular mechanisms of toxicity and growth inhibition of methanolic extracts were done using plants from only one population (2729 m a.s.l). We found that relative consumption rate (RCR), relative growth rate (RGR, Fig. 5), efficiency of conversion of ingested food (ECI) and efficiency of conversion of digested food (ECD) decreased with increasing concentration of extract (*P* < 0.001 in all cases) but there was no effect on approximate digestibility (AD)—see **SupportingInformation** for more details of the results – File 4.

### Indirect defences—volatile organic compounds

**Figure 6.**
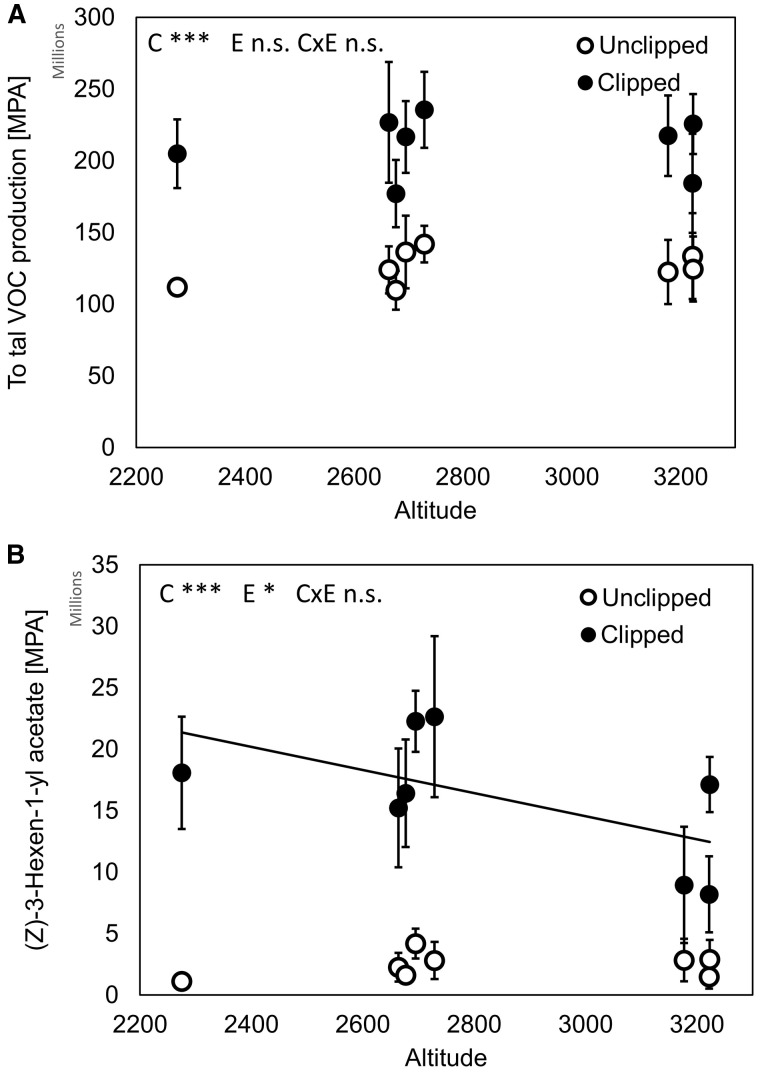
Effect of clipping (simulated herbivore treatment) and altitude on production of (A) total VOCs and (B) (Z)-3-Hexen-1-yl acetate. C, E and C × E indicate effects of clipping treatment, altitude and their interaction, respectively. **P < 0.01; ***P < 0.001; n.s. non-significant. MPA = mean peak area. Population means and SE are shown.

While there was no overall significant effect of altitude on VOC production, clipping treatment explained 22.5% of variability in the data (*F*_1,1168 _=_ _11.9, *P* = 0.002, Fig. 6A). Among 30 VOCs responsible for most variability in the data caused by clipping treatment, there were five heterocyclic compounds, 11 terpens, 11 alcohols, aldehydes or ketones and four esters. VOC production was increased due to clipping treatment 5–144 times compared to unclipped treatment for these compounds. VOCs most strongly affected by clipping were Valencene, (Z)-Linalool oxide (furanoid) and 5-Ethyl-2(5H)-furanone (Table 3). VOCs with mean highest production were (E)-2-Hexenal, (Z)-3-Hexen-1-yl acetate and (Z)-3-Hexen-1-ol (Table 3). Production of four of the 30 VOCs affected by clipping were correlated with altitude ((Z)-3-Hexen-1-yl acetate, *F*_1,18 _=_ _5.77, *P* = 0.03; (Z)-3-Hexenyl isovalerate, *F*_1,18 _=_ _3.97, *P* = 0.06; Caryophyllene (E), *F*_1,18 _=_ _3.49, *P* = 0.08; δ-Cadinene, *F*_1,18 _=_ _5.88, *P* = 0.03) with plants from lower altitudes producing more VOCs compared to plants from higher altitudes (Fig. 6B and Table 2). Clipped plants produced 3.5, 5.7, 3.5 and 4.3% less of (Z)-3-Hexen-1-yl acetate, (Z)-3-Hexenyl isovalerate, Caryophyllene (E) and δ-Cadinene per each 100 m of altitudinal increase.

**Table 3.** List of 30 VOCs responsible for most variability in the data caused by clipping treatment in the multivariate RDA analysis (first axis explained 22.5% of variability in the data; *F* = 11.9, *P* = 0.002). Class = class of *S. nubivola* VOC emission, MPA = mean peak area of specific compound recorded at chromatogram used as approximation of VOCs production (relative units), MPA clip = mean peak area of specific compound recorded at chromatogram of clipped plants, MPA control = mean peak area of control plants, Clip:not clip = ratio of MPA at clipped and not clipped plants, RDA scores = value on the first axis in RDA analyses, Population/Altitude = effect of population/altitude on production of particular compound.

Compound	Class	MPA clip × 10^3^	MPA control × 10^3^	Clip: not clip	RDA scores	Population	Altitude

2-Ethylfuran	H	976	27	35	0.941	n.s.	n.s.
(Z)-3-Hexen-1-ol	A	15074	386	39	0.934	n.s.	n.s.
(E)-2-Hexenal	A	19431	577	34	0.931	n.s.	n.s.
(Z)-2-Hexenal	A	394	11	35	0.882	n.s.	n.s.
5-Ethyl-2(5H)-furanone	H	707	9.5	75	0.846	n.s.	n.s.
(E,E)-2,4-Hexadienal	A	1103	63	17	0.802	n.s.	n.s.
Thymol	T,A	13	1.9	7	0.767	n.s.	n.s.
(Z)-3-Hexen-1-yl acetate	E	16112	2398	7	0.749	n.s.	0.03 (-)
(Z)-3-Hexenyl valerate	E	60	1.5	39	0.741	n.s.	n.s.
(E)-4-Oxohex-2-enal	H	2757	194	14	0.736	n.s.	n.s.
1,4-Cyclohex-2-enedione	A	95	7.7	12	0.729	n.s.	n.s.
(Z)-3-Hexenyl isovalerate	E	99	4.1	24	0.719	n.s.	0.06 (-)
cis-Thujane-4-ol	T	17	0.3	57	0.718	n.s.	n.s.
à-Terpineol	T,A	310	45	7	0.717	n.s.	n.s.
β-Copaene	T	324	7.6	42	0.699	n.s.	n.s.
3-Pentanone	A	233	23	10	0.695	n.s.	n.s.
(Z)-2-Penten-1-ol	A	358	10	34	0.687	n.s.	n.s.
(Z)-Linalool oxide (furanoid)	H,T	118	1.5	80	0.685	0.09	n.s.
2-Ethyl-1H-pyrrole	H	39	4.7	8	0.669	n.s.	n.s.
1-Hexanol	A	691	45	15	0.661	n.s.	n.s.
β-Thujene	T	33	4.3	8	0.660	n.s.	n.s.
δ-Cadinene	T	715	10	69	0.653	n.s.	0.03 (-)
β-Elemene	T	320	13	23	0.647	n.s.	n.s.
Valencene	T	1459	10	144	0.628	0.08	n.s.
Cumenol	A	24	4.7	5	0.624	n.s.	n.s.
Caryophyllene (E)	T	123	8.4	15	0.613	n.s.	0.08 (-)
1-Octen-3-yl acetate	E	384	56	7	0.597	0.08	n.s.
C15 branched hydrocarbon	U	126	21	6	0.595	n.s.	n.s.
Unknown compound MW 236	U	20	0.3	71	0.593	n.s.	n.s.
α-Amorphene	T	171	3.2	53	0.565	n.s.	n.s.
Total VOCs production		210932	125421	1.7		n.s.	n.s.

n.s. = non-significant, number in the population/altitude columns indicates *P* value from test of relationship between particular VOC and population/altitude.

(-) indicates lower VOC production in higher compared to lower altitude.

H: heterocyclic compounds; T: terpens; A: alcohols, aldehydes or ketones; E: esters.

### Correlations among growth and defence strategies

**Figure 7.**
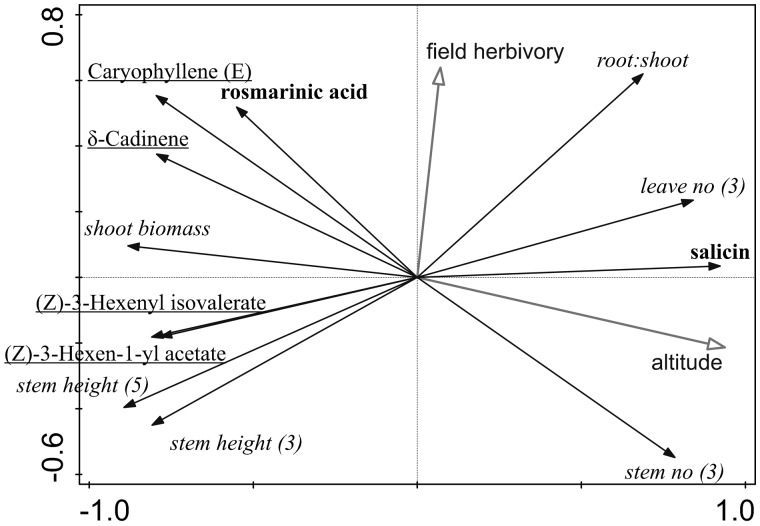
Diagram indicating relationship among plant growth and defence traits (PCA analysis). First axis explains 66.25% of variability in the data and second axis additional 14.68%. Altitude and field herbivory were added as supplementary variables and were not thus part of the analysis. Stem no (3) indicates number of stems after three months growth. Traits within growth and two defence strategies significantly correlated with altitude (direct defence and indirect defence) were in italics, bold and underlined, respectively.

When we compared all plant growth and defence traits correlated with altitude in multivariate PCA analysis, the first axis explained 66.25% of the variability in the data and the second axis added 14.68% (Fig. 7). While we plotted only plant defence traits correlated with altitude, the different traits are largely uncorrelated (Fig. 7, see **SupportingInformation** for more details of results – File 5). The exceptions were the negative correlations between salicin content and most of the growth traits (see **SupportingInformation** for more details of the results – File 3).

## Discussion

The study described a wide range of anti-herbivore defence strategies in *Salvia nubicola* and demonstrated that most of these strategies are correlated with altitude. Changing intensity of plant–herbivore interactions along the altitudinal gradient relating to future climate change may thus have strong effects on performance of the plants. Our work is one of the first studies simultaneously measuring variation in plant growth, direct and indirect defences as well as tolerance to tissue loss as a consequence of herbivore attack along altitudinal gradient in populations of a single wide spread species. We found that plants originating from lower altitudes, where they experience higher herbivore pressure, differed from plants from higher altitudes in growth rate as well as in production of phenolic compounds (direct defence) and production of VOCs (indirect defence). However, there were no differences in tolerance to herbivory and effect of methanolic extracts on generalist herbivore *Spodoptera littoralis* (direct defences) among populations distributed along the altitudinal gradient.

In agreement with other studies (Bruelheide and Scheidel 1999; Rasmann *et al.* 2014*b*; Pellissier *et al.* 2014), we also found a strong negative relationship between altitude and herbivore damage recorded in populations of *S. nubicola*. Moreover, differences in herbivore damage recorded in our study system ranged from 5% to 35% providing us with a good model system for testing our hypotheses.

We recorded higher growth of plants originating from lower compared to higher altitudes. These differences correspond to other studies comparing plant traits along altitudinal gradients and could be explained by adaptation to harsh climate conditions in higher altitudes (reviewed by Núñez-Farfán and Schlichting 2001). Increased growth of plants from lower altitudes might also enable plants to withstand herbivore damage as predicted by the resource availability hypothesis (Coley *et al.* 1985). It hypothesizes that species growing in more suitable habitats with higher recourse availability are adapted to these conditions by increased growth rate by which they can better deal with higher herbivore attack.

Jamieson *et al.* (2012) suggested that climate change will also inﬂuence plant tolerance to insect damage. For example, Huttunen *et al.* (2007) reported that silver birch trees have a high capacity to tolerate defoliation and that under the combination of warmer temperatures and elevated CO_2_, defoliated trees grew better than undefoliated controls. Because elevated temperatures both increase net primary production and extend the growing season, warming may improve the ability of plants to compensate for defoliation. That is why we also expected differences in tolerance to leaf damage at *S. nubicola* between populations along the altitudinal gradient. However, altitude had no effect on plant differences in plant growth between clipped and unclipped plants indicating no adaptation in tolerance to tissue loss in our system. An explanation could be that we simulated herbivore damage by mechanical clipping because our original plan with generalist herbivore *S. littoralis* failed. However, many studies showed that clipping has very similar effects to herbivore damage (McNaughton 1983; van Kleunen *et al.* 2004; Frye *et al.* 2012; Moreira *et al.* 2012; Scheepens *et al.* 2015). Moreover, production of VOCs was much increased after clipping treatment and clipping could be thus considered as effective simulation of herbivore damage at least in case of VOC production. The second reason could be the low impact of herbivore damage on plant performance and thus no selection on plant tolerance to tissue loss. Although we have recorded up to 35% of leaf damage (population mean) as a result of herbivore attack in the field, the effects of this leaf damage on plants fitness are not known. It could also be the case that tolerance is not so important for *S. nubicola* and it uses other defence mechanisms such as production of secondary metabolites or VOCs or it rather invests to increased growth.

Salicin and rosmarinic acid are well known defence phenolic secondary metabolites against herbivores and infections and were shown to cause biological activity of several species including *Salvia* (Tegelberg and Julkunen-Tiitto 2001; Petersen *et al.* 2009; Boeckler *et al.* 2011). In agreement with our hypothesis, we recorded higher production of rosmarinic acid at plants from lower altitudes but an opposite pattern was found for salicin production. The reason could be that both abiotic and biotic stresses contribute to shaping secondary chemistry phenotypes. Phenolic compounds can simultaneously reduce insect performance and protect leaves from photo-damage by acting as antioxidants (Close and McArthur 2002; Forkner *et al.* 2004; Rasmann *et al.* 2014*c*). Both compounds (salicin and rosmarinic acid) belong to strong antioxidants and can protect plants against oxidative stress caused by higher intensity of UV radiation at higher altitude (Warren *et al.* 2003; Taremi *et al.* 2015; Vidović *et al.* 2015). Photo-protective function of salicin thus seems to prevail in the case of *S. nubicola*. Moles *et al.* (2011) also proposed that climate affects the relative cost of losing leaf tissue damaged by herbivores, being more costly in harsh and unproductive environments (Coley *et al.* 1985). This can thus result in different selective regimes along climatic gradient. Selection pressure due to herbivory may be thus greater in harsher climate in higher altitudes even if the herbivory rate is lower than in a warm productive climate (Johnson and Rasmann 2011). Higher content of salicin (direct defence) found in higher altitudes could thus be result of such higher selection pressure.

Although levels of some phenolic compounds in fact increase rather than decrease with increasing altitude because of their photo-protective function, the levels of VOCs typically decreases (Zvereva and Kozlov 2006; Bidart-Bouzat and Imeh-Nathaniel 2008). This might be an adaptation for higher herbivore pressure in lower altitudes. Although *S. nubicola* in our experiment increased production of VOCs after clipping treatment, we have not recorded any effect of altitude on total VOC production as expected and shown in other studies (Zvereva and Kozlov 2006; Bidart-Bouzat and Imeh-Nathaniel 2008; Wason *et al.* 2013). However, recent study on *Vicia sepium* (Rasmann *et al.* 2014*b*) indicated that this pattern is not general. Although total VOCs production was not correlated with altitude, two green leaf volatile esters [(Z)-3-Hexen-1-yl acetate and (Z)-3-Hexenyl isovalerate] were produced more by *S. nubicola* plants from lower altitudes. These compounds are commonly emitted from mechanically and herbivore damaged plants and may serve as attractants to predators and parasitoids of herbivores and induce several important plant defence pathways (Kost and Heil 2006; D’Auria *et al.* 2007). The same is valid also for two sesquiterpenes [δ-Cadinene and Caryophyllene (E)], the production of which was also increased in lower altitudes and which were found to be involved in indirect plant defence (Rasmann *et al.* 2005; Frost *et al.* 2007).

We also found that the methanolic extracts of *S. nubicola* directly affected feeding and growth of *S. littoralis* larvae and, in higher doses, caused chronic toxicity. It is in concordance with previous studies (Pavela 2012; Sajfrtova *et al.* 2013; Pavela 2014; Pavela *et al.* 2014), which also found toxic effect of compounds obtained from Lamiaceae family plants against insects. Although this effect was quite strong, it did not differ among populations originating from different altitudes. It therefore does not represent an adaptation to changing herbivore pressure along altitudinal gradient. An alternative explanation for the lack of correlation between altitude and effect of *S. nubicola* extracts on *S. littoralis* is that *S. nubicola* is not attacked by generalist but specialized herbivores in its natural populations. It is possible that we would get different results when testing the effect of extracts on real herbivores in the field (Ali and Agrawal 2012). Alternatively, specialized herbivores could be adapted to compounds produced by *S. nubicola* and higher herbivore damage found in natural populations in the field could be a consequence of more suitable climatic and habitat conditions for particular insect species.

Observed patterns for the different defence traits could have been different if the data were collected from plants directly in the field. Range of both biotic and abiotic factors could affect plant defence systems of the plants growing in the field along altitudinal gradient. Rasmann *et al.* (2014*c*) suggest that plants from higher altitudes may have developed tougher leaves as an adaptation to severe climatic conditions, but this may indirectly confer increased resistance to herbivores. The selective forces of abiotic conditions (e.g. cold hardiness) might thus be stronger than biotic ones (e.g. resistance to herbivores) along altitudinal gradients. It could be thus useful to perform further experiments including measuring defence strategies in combination with field transplant experiment when plants could experience real herbivore damage under real climatic conditions.

Rasmann *et al.* (2014b) also suggested that the potentially low-cost inducible defence strategy may be particularly suitable in environments where the herbivore composition and abundance are highly variable in space and time (such as high altitudes) and where the costs of establishing constitutive defence may be too high due to low productivity of the environment (Agrawal and Karban 1999). It would mean that there is a trade-off between constitutive direct defences (production of phenolic compounds in our study) and induced indirect defences (production of VOCs in our study) along altitudinal gradient (Ballhorn *et al.* 2008). However, it was not the case in our study since there was consistent negative effect of altitude on production of VOCs.

Finally, it should be noted that VOC production may also be herbivore-specific and that induction may vary in response to attacks by different herbivores (Pichersky *et al.* 2006). However, here, we show that both constitutive direct resistance and VOC production (indirect defences) are reduced at high elevation. Real herbivores could cause bigger differences but we do not suppose that the pattern could be completely opposite.

## Conclusions

Our study is one of the first comparing plant growth, tolerance to tissue loss, direct defences including production of phenolic compounds and effect of methanolic extracts on generalist herbivore larvae and indirect defences including production of VOCs. Although we found that *S. nubicola* developed a range of defence strategies, they do not seem to be used simultaneously in all populations even though most of them are correlated with altitude. It is in agreement with current knowledge that co-expression of multiple defences might be costly for a plant, since investment in defensive traits is assumed to reduce the resource availability for growth and reproduction (Heil and Bostock 2002; Koricheva *et al.* 2004; Ballhorn *et al.* 2008). Constraints on simultaneous resource allocation to multiple defensive strategies thus result in trade-offs among different types of defence strategies. Our study thus shows an importance of simultaneous study of different defence strategies since understanding these trade-offs could be necessary for detecting the mechanisms allowing plants to cope with future climate change.

## Sources of Funding

Our work was funded by Czech Science Foundation (project no. 13-10850P). T.D., M.R. and Z.M. were also supported by a long-term research development project no. RVO 67985939 and institutional project MŠMT. J.S. was supported by a grant from the Czech Ministry of Agriculture (Mze ČR) RO0416.

## Contributions by the Authors

T.D., M.R. and Z.M. wrote the manuscript and designed the experiments; J.S., P.M., J.R. and R.P. commented the manuscript; T.D. and M.R. collected field and greenhouse growth data; J.S. designed and collected data on experiment with *S. littoralis* in the greenhouse, P.M. designed and collected data on phenolic compounds, J.R. designed and collected data on volatile organic compounds and R.P. designed and collected data on effect of methanolic extracts on *S. littoralis*. T.D. and Z.M. conducted the statistical analyses.

## Conflict of Interest Statement

None declared.

## Supplementary Material

Supplementary Data

## Supporting Information

The following additional information is available in the online version of this article –

**File 1.** Details of analyses of phenolic compounds produced by *S. nubicola*.

**File 2.** Details of analyses of volatile organic compounds produced by *S. nubicola*.

**File 3.** Table. Detailed test results of effect of population, altitude and clipping and their interaction on plant traits (number of stems, number of leaves, height of the longest stem, root, shoot, root:shoot and total dry biomass weight).

**File 4.** Table. Detailed test results of effect of methanolic extracts from *S. nubicola* plants on generalist herbivore *Spodoptera littoralis* along altitudinal gradient.

**File 5.** Table. Correlation matrix of defence strategies of *S. nubicola*.
