# Supplementary material for: Trade-off among different anti-herbivore defence strategies along an altitudinal gradient
Source: AoB Plants. 2016 Jul 11;8:plw026. doi: 10.1093/aobpla/plw026 (PMC4940502; doi:10.1093/aobpla/plw026)
Supplement: Supplementary Data [file supp_8_plw026_index.html]

Supplementary Data 

# Trade-off among different anti-herbivore defence strategies along an altitudinal gradient

## Supplementary Data

files

- Supplementary Data - zip file
