# Supplementary material for: Trade-off among different anti-herbivore defence strategies along an altitudinal gradient
Source: AoB Plants. 2016 Jul 11;8:plw026. doi: 10.1093/aobpla/plw026 (PMC4940502; doi:10.1093/aobpla/plw026)
Supplement: Supplementary Data [file supp_plw026_suppl_data.zip › aobplants-15357-s07.docx]

# OPEN ACCESS – RESEARCH ARTICLE

# Trade-off among different anti-herbivore defence strategies along an altitudinal gradient

### Tomáš Dostálek ^1,2,^*, Maan Bahadur Rokaya ^1^, Petr Maršík ^3^, Jan Rezek ^3^, Jiří Skuhrovec ^4^, Roman Pavela ^4^, Zuzana Münzbergová ^1,2^

^1^Institute of Botany, The Czech Academy of Sciences, Zámek 1, Průhonice, CZ-25243, Czech Republic.

^2^Department of Botany, Faculty of Science, Charles University in Prague, Benátská 2, Prague, CZ-12801, Czech Republic.

^3^Institute of Experimental Botany, The Czech Academy of Sciences, Rozvojová 313, Prague, CZ-16502, Czech Republic.

^4^Crop Research Institute, Drnovská 507, Prague, CZ-16106, Czech Republic.

*Corresponding author

Corresponding author’s e-mail address: tomas.dostalek@gmail.com

Running head: Trade-off among anti-herbivore defences along an altitudinal gradient
