# Supplementary material for: Trade-off among different anti-herbivore defence strategies along an altitudinal gradient
Source: AoB Plants. 2016 Jul 11;8:plw026. doi: 10.1093/aobpla/plw026 (PMC4940502; doi:10.1093/aobpla/plw026)
Supplement: Supplementary Data [file supp_plw026_suppl_data.zip › aobplants-15357-s01.docx]

**Supporting information: File 1**

**Analyses of phenolic compounds**

Sampled leaves were deep frozen in -80°C until extraction in June 2015. Frozen leaves were ground to a fine powder with mortar and pestle under liquid nitrogen. Ground plant material (0.3 g) was moved into 2 mL plastic tube and 1.5 mL of 80% MeOH was added. The sample was extracted for 3 min in ultrasonic bath and then mixed for 20 min on a rotational tube mixer (Revolver H5600, Labnet, Edison, USA). After the extraction, the sample was centrifuged for 1 min at 13,000 rpm (Micro 20, Hettich, Germany), the upper liquid fraction (crude extract) was removed by pipette to the plastic tube. The pellet was resuspended by the same volume of 80% MeOH and subsequently extracted once more by the same procedure.

The combined extracts were evaporated to dryness by the stream of nitrogen, dissolved in deionized water (1 mL) and applied on to SPE 96-well plate filled by Oasis® MCX sorbent 30 µm (30 mg, Waters, USA) preconditioned with 1 mL of MeOH and washed with 2 mL of deionized water. Measured compounds were eluted with 3 volumes of 300 µL of pure MeOH and evaporated under a stream of nitrogen and stored in -80°C until analysis. Immediately before the analysis, the samples were dissolved with mobile phase and applied to LC/MS/MS.

Selected secondary metabolites were determined using tandem mass spectrometer Q-Trap 4000 (AB Sciex, USA) equipped with liquid chromatography system Ultimate 5000 (Dionex, USA). Kinetex Phenyl-hexyl column (100 x 2.10mm, 1.7µm, 100 A, Phenomenex, USA) and mobile phase water (A) and MeOH (B) both with addition of 5mM ammonium formate and 0.25% of formic acid (v/v) was used for all analysed compounds. Two different analytical methods were developed depending on the type of analytes which were both subsequently applied on each measured sample. The first method was developed for detection of 8 coumarins and their glycosides (esculin, esculetin, scopoletin, 8-methoxycoumarin, isoscopoletin, henriarin, umbelliferone, isofraxidin) with gradient elution started as isocratic for 1 min at 20% B. Then consecutively ramped to 80% and 100% B in 8 and 9 min, isocratic to 13 min and then returned at starting concentration (20% B) in 14 min followed by equilibration for 5 min. Column temperature was set at 40°C. The second method was optimized for quantitation of other phenolic compounds including salicin, rosmarininic acid and lignans (arctigenin, arctiin, lariciresinol, pinoresinol, nortrachelogenin, secoisolariciresinol and matairesinol). The elution gradient for the second method was kept at 20% B for 1 min, then linear to 100% B in 6 min, isocratic to 9 min and equilibrated from 10 to 14 min at 20% B. Column temperature was 35°C. The flow rate of the mobile phase (0.25 mL/min) and sample injection volume (5 µL) were the same for both methods. Analysis was performed using multiple reaction monitoring (MRM) in positive ion mode with ionization parameters optimized for each analyte. For the quantitation, the data processing was carried out using MultiQuant 2.0.2 software (AB Sciex, USA). The amount of the found compounds was determined by method of external standards.
