# Supplementary material for: Trade-off among different anti-herbivore defence strategies along an altitudinal gradient
Source: AoB Plants. 2016 Jul 11;8:plw026. doi: 10.1093/aobpla/plw026 (PMC4940502; doi:10.1093/aobpla/plw026)
Supplement: Supplementary Data [file supp_plw026_suppl_data.zip › aobplants-15357-s02.docx]

**Supporting information: File 2**

**Analyses of volatile organic compounds**

VOCs were trapped in Tenax TA sorbent tubes (Gerstel GmbH & Co. KG, Mülheim an der Ruhr, Germany) from 2 L of air under the flow of 100 mL/min using Casella Cel Apex Air Sampling Pump equipped with Low Flow Adaptor (Casella CEL, Stokenchurch, UK). Analysis was performed on LECO Pegasus 4D GC×GC-TOFMS system (Leco Corporation, St. Joseph, MI, USA) containing Agilent 7890 gas chromatograph (Agilent technologies, Santa Clara, CF, USA) equipped with a LECO quad-jet dual stage thermal modulator; Gerstel MultiPurpose Sampler (MPS), Gerstel Thermal Desorption Unit (TDU) and temperature programmed CIS4 inlet (Gerstel GmbH & Co. KG, Mülheim an der Ruhr, Germany). Gas chromatograph was fitted with precolumn - Restek Siltek Deactivated Guard Column (2 m × 0.25 mm I.D.); primary column - SGE Analytical Science BPX-5, non-polar, 5% phenyl polysilphenylene-siloxane (30 m × 0.25 mm I.D. × 0.5 µm film), modulatory column - SGE Analytical Science BPX-5 (0.1 m × 0.25 mm I.D. × 0.5 µm film), secondary column - SGE Analytical Science BPX-50, moderately-polar, 50% phenyl polysilphenylene-siloxane (1.447 m × 0.1 mm I.D. × 0.1 µm film) and transfer line - SGE Analytical Science BPX-50 (0.21 m × 0.1 mm I.D. × 0.1 µm film). Helium BIP (purity 5.7) was used as carrier gas (Air Products, Decin, Czech Republic).

Trapped VOCs were desorbed from Tenax TA tubes in TDU under following conditions: initial temperature 20°C; delay time 1 min; temperature ramp 250°C/min up to 260 °C; hold time 20 min; transfer temperature in Fixed mode at 270°C; splitless desorption mode; Retain tube – Standby cooling mode at 260°C; helium flow 50 mL/min. VOCs were then trapped in CIS4 cooled inlet with baffled liner. Inlet was operated in solvent vent mode under following conditions: purge time 300 s; purge flow 200 mL/min; solvent vent time 0.6 s; solvent vent flow 50 mL/min; solvent vent pressure 5 psi, purge time 5 min, purge flow 200 mL/min; initial temperature -20°C; equilibration time 1 min; initial time 1 min; temperature ramp 10°C/s up to 260°C; hold time 30 min. The analysis was then performed under the following conditions: helium at constant flow 1 mL/min; primary oven temperature 35°C (5 min hold), temperature ramp 5°C/min until 330 °C (5 min hold); modulation period 3 s; 0.75 s hot pulse; 0.75 s cold pulse; modulator temperature program 30°C above primary column program; chiller at -80°C; secondary column operated 0°C above primary column program; transfer line at 280°C. Time of flight mass detector (EI) was operated under following conditions: ion source at 250°C; acquisition delay 180 s; spectra collected between 29-600 amu at 100 Hz; detector voltage 1525 V and electron energy -70 V. Identification of compounds was based on comparison of their spectra with those of mass libraries (NIST, LECO/Fiehn Metabolomics Library), comparison of retention indices (RI) with those from literature and comparison of retention times and mass spectra with those of pure analytical standards available. Alignment of peaks and comparison of samples was done using the Statistical Compare module integrated in ChromaTOF software (Leco Corporation, St. Joseph, MI, USA).
