# Supplementary material for: Trade-off among different anti-herbivore defence strategies along an altitudinal gradient
Source: AoB Plants. 2016 Jul 11;8:plw026. doi: 10.1093/aobpla/plw026 (PMC4940502; doi:10.1093/aobpla/plw026)
Supplement: Supplementary Data [file supp_plw026_suppl_data.zip › aobplants-15357-s03.docx]

**Supporting information: File 3**

Effect of population and altitude on plant traits (number of stems, number of leaves and height of longest stem) of 3 months old plants of *S. nubicola* in July 2014. In the beginning of August, herbivory was simulated by clipping. Effect of population, altitude and clipping and their interaction on plant traits (number of stems, number of leaves, height of the longest stem, root, shoot, root:shoot and total dry biomass weight) in the end of experiment - September 2014. Population and altitude were recorded on the localities where seeds were collected. Tests were done using GLM, n=368). Ef.=effect on plant trait when “+” indicates positive effect of higher altitude, i.e. plants in higher altitudes produce more stems. “-” indicates negative effect of higher altitude, i.e. plants in higher altitudes produce fewer stems. *P<0.05; **P<0.01; ***P<0.001; n.s. non-signiﬁcant.

| Effect of / Effect on |  | Stem no | | | | Leave no | | | | Stem height | | | | Shoot weight | | | | Root weight | | | | Root:shoot | | | | Total biomass weight | | | |
| --- | --- | --- | --- | --- | --- | --- | --- | --- | --- | --- | --- | --- | --- | --- | --- | --- | --- | --- | --- | --- | --- | --- | --- | --- | --- | --- | --- | --- | --- |
|  | Df | F | R^2^ | P | Ef. | F | R^2^ | P | Ef. | F | R^2^ | P | Ef. | F | R^2^ | P | Ef. | F | R^2^ | P | Ef. | F | R^2^ | P | Ef. | F | R^2^ | P | Ef. |
| **Growth – 3 month** | | |  |  |  |  |  |  |  |  |  |  |  |  |  |  |  |  |  |  |  |  |  |  |  |  |  |  |  |
| Population | 20 | 4.23 | 0.29 | 0.000 |  | 3.86 | 0.18 | 0.000 |  | 3.70 | 0.17 | 0.000 |  |  |  |  |  |  |  |  |  |  |  |  |  |  |  |  |  |
| Altitude | 1 | 35.12 | 0.12 | 0.000 | + | 11.47 | 0.03 | 0.000 | - | 11.03 | 0.03 | 0.000 | - |  |  |  |  |  |  |  |  |  |  |  |  |  |  |  |  |
| **Growth – 5 month** |  |  |  |  |  |  |  |  |  |  |  |  |  |  |  |  |  |  |  |  |  |  |  |  |  |  |  |  |  |
| Population | 20 | 1.96 | 0.24 | 0.000 |  | 1.02 | 0.11 | 0.444 |  | 3.04 | 0.27 | 0.000 |  | 1.94 | 0.19 | 0.013 |  | 1.51 | 0.16 | 0.083 |  | 2.01 | 0.20 | 0.009 |  | 1.75 | 0.18 | 0.031 |  |
| Altitude | 1 | 0.01 | 0.00 | 0.931 |  | 2.70 | 0.01 | 0.102 |  | 16.08 | 0.08 | 0.000 | - | 12.35 | 0.06 | 0.000 | - | 0.16 | 0.00 | 0.687 |  | 6.51 | 0.03 | 0.012 | + | 8.75 | 0.05 | 0.004 | - |
| **Response to clipping** | | |  |  |  |  |  |  |  |  |  |  |  |  |  |  |  |  |  |  |  |  |  |  |  |  |  |  |  |
| Clipping | 1 | 17.52 | 0.05 | 0.000 | - | 34.45 | 0.07 | 0.000 | + | 15.20 | 0.01 | 0.000 | + | 9.24 | 0.01 | 0.003 | - | 7.58 | 0.02 | 0.006 | - | 0.96 | 0.00 | 0.328 |  | 1.69 | 0.00 | 0.194 |  |
| Population | 20 | 1.06 | 0.06 | 0.381 |  | 0.62 | 0.04 | 0.558 |  | 4.64 | 0.07 | 0.000 |  | 1.65 | 0.05 | 0.040 |  | 1.59 | 0.08 | 0.053 |  | 2.89 | 0.11 | 0.000 |  | 0.85 | 0.03 | 0.649 |  |
| Population × clip. | 20 | 1.38 | 0.08 | 0.118 |  | 1.53 | 0.06 | 0.068 |  | 1.29 | 0.02 | 0.178 |  | 0.75 | 0.02 | 0.773 |  | 1.27 | 0.07 | 0.193 |  | 0.85 | 0.03 | 0.648 |  | 1.27 | 0.05 | 0.199 |  |
| Clipping | 1 | 17.22 | 0.05 | 0.000 | - | 33.74 | 0.07 | 0.000 | + | 12.83 | 0.01 | 0.000 | + | 9.69 | 0.02 | 0.002 | - | 7.13 | 0.02 | 0.008 | - | 0.81 | 0.00 | 0.369 |  | 1.73 | 0.00 | 0.189 |  |
| Altitude | 1 | 1.67 | 0.01 | 0.167 |  | 2.48 | 0.01 | 0.116 |  | 32.12 | 0.03 | 0.000 | - | 8.78 | 0.01 | 0.003 | - | 0.91 | 0.00 | 0.341 |  | 12.16 | 0.03 | 0.000 | + | 1.85 | 0.00 | 0.174 |  |
| Altitude × clip. | 1 | 0.29 | 0.00 | 0.588 |  | 0.00 | 0.00 | 0.970 |  | 0.90 | 0.00 | 0.343 |  | 0.73 | 0.00 | 0.394 |  | 1.83 | 0.00 | 0.177 |  | 0.64 | 0.00 | 0.425 |  | 2.77 | 0.01 | 0.097 |  |
