# Supplementary material for: Trade-off among different anti-herbivore defence strategies along an altitudinal gradient
Source: AoB Plants. 2016 Jul 11;8:plw026. doi: 10.1093/aobpla/plw026 (PMC4940502; doi:10.1093/aobpla/plw026)
Supplement: Supplementary Data [file supp_plw026_suppl_data.zip › aobplants-15357-s04.docx]

**Supporting information: File 4**

**Detailed test results of effect of methanolic extracts from *S. nubicola* plants on generalist herbivore *Spodoptera littoralis* along elevation gradient.**

The lethal doses (LD_50_, LD_90_) and feeding deterrence index (FDI) and their 95% confidence intervals (CL_95_) for third instar *S. littoralis* larvae were calculated for methanolic extracts from plants from 5 *S. nubicola* populations along altitudinal gradient using the Spearman-Karber method with Abbot correction (Hamilton et al., 1977).

| Locality | Elevation | LD_50_ (CI95)  µg.cm^-2^ | LD_90_ (CI95)  µg.cm^-2^ | Chi | FDI (CI95)  LD_50_ µg.cm^-2^ | FDI (CI95)  LD_90_ µg.cm^-2^ | Chi |
| --- | --- | --- | --- | --- | --- | --- | --- |
| DA | 2275 | 162 (139-211) | 521 (423-546) | 0.985 | 128 (123-136) | 199 (182-211) | 0.856 |
| TI2 | 2664 | 158 (133-196) | 515 (472-558) | 0.325 | 125 (119-129) | 189 (178-201) | 0.256 |
| CH2 | 2729 | 122 (103-157) | 425 (396-493) | 1.256 | 112 (98-139) | 178 (156-189) | 0.355 |
| DP1 | 3214 | 133 (96-145) | 598 (515-612) | 1.569 | 132 (125-156) | 201 (172-225) | 1.727 |
| PIS3 | 3262 | 175 (138-228) | 586 (529-623) | 1.598 | 132 (129-139) | 212 (195-233) | 0.328 |

The effect of crude extract of *S. nubicola* plants from locality at 2729 m a.s.l. incorporated into larval diet on food consumption and utilization (mean ± SE) by *S. littoralis* larvae. The extracts were presented in an artificial diet for 10 days.

| Plant extract (mg g^-1^) | RCR (mg.mg.day^-1^) | RGR (mg.mg.day^-1^) | ECI (%) | ECD (%) | AD (%) |
| --- | --- | --- | --- | --- | --- |
| 0 | 15.32 ± 0.31^a^ | 5.32 ± 0.35^d^ | 31.26 ± 0.29^d^ | 38.99 ± 3.18^d^ | 50.84 ± 3.22 |
| 0.5 | 19.25 ± 1.22^c^ | 4.72 ± 0.43^cd^ | 22.56 ± 1.05^c^ | 31.25 ± 3.19^b^ | 48.12 ± 5.26 |
| 1.0 | 21.18 ± 1.15^e^ | 4.25± 0.32^c^ | 19.33 ± 1.03^a^ | 22.35 ± 1.96^a^ | 45.22 ± 5.22 |
| 1.6 | 25.35 ± 1.76^b^ | 3.24 ± 0.39^b^ | 13.92 ± 0.65^b^ | 19.23 ± 2.18^c^ | 47.57 ± 3.11 |
| 2.5 | 31.11 ± 1.55^d^ | 1.75 ± 0.31^a^ | 12.78 ± 1.35^a^ | 17.85 ± 1.42^a^ | 48.43 ± 2.34 |
| *P* | 0.0001 | 0.0001 | 0.0001 | 0.0001 | N.S. |
| *F* | 232.11 | 129.22 | 327.94 | 541.21 | 0.356 |

RCR, relative consumption rate (mg/mg body weight/day); RGR, relative growth rate; ECI, efficiency of conversion of ingested food; ECD, efficiency of conversion of digested food; AD, approximate digestibility. Mean values with the same superscript letter are not significantly different (p≤0.05).
