# Supplementary material for: Trade-off among different anti-herbivore defence strategies along an altitudinal gradient
Source: AoB Plants. 2016 Jul 11;8:plw026. doi: 10.1093/aobpla/plw026 (PMC4940502; doi:10.1093/aobpla/plw026)
Supplement: Supplementary Data [file supp_plw026_suppl_data.zip › aobplants-15357-s05.docx]

**Supporting information: File 5**

Correlation matrix of defence strategies of *S. nubicola*. Numbers is the table are correlation coefficients, number of pairwise comparison (N) and P values^#^.

|  |  |  |  | Growth | | | | | | | Phenolic compounds | | | | | VOCs | | | | | |
| --- | --- | --- | --- | --- | --- | --- | --- | --- | --- | --- | --- | --- | --- | --- | --- | --- | --- | --- | --- | --- | --- |
|  |  | Altitude | Field herbivory | Stem no 3M | Height 3M | Leave no 3M | Height 5M | Shoot weight | Root:shoot | Salicin | | Rosm.  acid | Esculin | Esculetin | 196 analyte | | 447 analyte | 630 analyte | 764 analyte | Total VOCs |  |
|  | Altitude | 1.0000 | -.6555 | .6351 | -.4532 | .4220 | -.5986 | -.6284 | .4676 | .9070 | | -.5313 | .0319 | -.0398 | -.5999 | | -.6085 | -.7083 | -.6257 | .0060 |  |
|  |  | N=27 | N=27 | N=21 | N=21 | N=21 | N=21 | N=21 | N=21 | N=8 | | N=8 | N=8 | N=8 | N=8 | | N=8 | N=8 | N=8 | N=8 |  |
|  |  | p= --- | p=.000 | p=.002 | p=.039 | p=.057 | p=.004 | p=.002 | p=.033 | p=.002 | | p=.175 | p=.940 | p=.925 | p=.116 | | p=.109 | p=.049 | p=.097 | p=.989 |  |
|  | Field herb | -.6555 | 1.0000 | -.6256 | .3193 | -.1234 | .3145 | .3529 | -.4584 | -.0254 | | .6681 | .4458 | .7466 | -.4498 | | .0333 | .3588 | .2080 | -.4018 |  |
|  |  | N=27 | N=27 | N=21 | N=21 | N=21 | N=21 | N=21 | N=21 | N=8 | | N=8 | N=8 | N=8 | N=8 | | N=8 | N=8 | N=8 | N=8 |  |
|  |  | p=.000 | p= --- | p=.002 | p=.158 | p=.594 | p=.165 | p=.117 | p=.037 | p=.952 | | p=.070 | p=.268 | p=.033 | p=.263 | | p=.938 | p=.383 | p=.621 | p=.324 |  |
| Growth | Stem no 3M | .6351 | -.6256 | 1.0000 | -.5324 | .2772 | -.6805 | -.7053 | .7197 | .8803 | | -.5030 | -.0301 | -.0600 | -.3256 | | -.5890 | -.6235 | -.3711 | .1120 |  |
|  |  | N=21 | N=21 | N=21 | N=21 | N=21 | N=21 | N=21 | N=21 | N=8 | | N=8 | N=8 | N=8 | N=8 | | N=8 | N=8 | N=8 | N=8 |  |
|  |  | p=.002 | p=.002 | p= --- | p=.013 | p=.224 | p=.001 | p=.000 | p=.000 | p=.004 | | p=.204 | p=.944 | p=.888 | p=.431 | | p=.124 | p=.099 | p=.365 | p=.792 |  |
|  | Height 3M | -.4532 | .3193 | -.5324 | 1.0000 | -.1518 | .8564 | .7884 | -.7338 | -.7581 | | .4618 | -.1494 | .0752 | .4557 | | .5565 | .4849 | .4207 | -.3720 |  |
|  |  | N=21 | N=21 | N=21 | N=21 | N=21 | N=21 | N=21 | N=21 | N=8 | | N=8 | N=8 | N=8 | N=8 | | N=8 | N=8 | N=8 | N=8 |  |
|  |  | p=.039 | p=.158 | p=.013 | p= --- | p=.511 | p=.000 | p=.000 | p=.000 | p=.029 | | p=.249 | p=.724 | p=.859 | p=.256 | | p=.152 | p=.223 | p=.299 | p=.364 |  |
|  | Leave no 3M | .4220 | -.1234 | .2772 | -.1518 | 1.0000 | -.3866 | -.2781 | .2905 | .3368 | | .1876 | .6212 | .3908 | -.3858 | | -.4814 | -.4680 | -.6057 | -.0746 |  |
|  |  | N=21 | N=21 | N=21 | N=21 | N=21 | N=21 | N=21 | N=21 | N=8 | | N=8 | N=8 | N=8 | N=8 | | N=8 | N=8 | N=8 | N=8 |  |
|  |  | p=.057 | p=.594 | p=.224 | p=.511 | p= --- | p=.083 | p=.222 | p=.201 | p=.415 | | p=.656 | p=.100 | p=.338 | p=.345 | | p=.227 | p=.242 | p=.112 | p=.861 |  |
|  | Height 5M | -.5986 | .3145 | -.6805 | .8564 | -.3866 | 1.0000 | .8587 | -.8060 | -.8555 | | .3537 | -.3653 | -.0638 | .6283 | | .8392 | .5508 | .3998 | -.0919 |  |
|  |  | N=21 | N=21 | N=21 | N=21 | N=21 | N=21 | N=21 | N=21 | N=8 | | N=8 | N=8 | N=8 | N=8 | | N=8 | N=8 | N=8 | N=8 |  |
|  |  | p=.004 | p=.165 | p=.001 | p=.000 | p=.083 | p= --- | p=.000 | p=.000 | p=.007 | | p=.390 | p=.374 | p=.881 | p=.095 | | p=.009 | p=.157 | p=.326 | p=.829 |  |
|  | Shoot weight | -.6284 | .3529 | -.7053 | .7884 | -.2781 | .8587 | 1.0000 | -.6473 | -.8927 | | .1365 | -.4209 | -.3541 | .4510 | | .5961 | .7411 | .5936 | -.1490 |  |
|  |  | N=21 | N=21 | N=21 | N=21 | N=21 | N=21 | N=21 | N=21 | N=8 | | N=8 | N=8 | N=8 | N=8 | | N=8 | N=8 | N=8 | N=8 |  |
|  |  | p=.002 | p=.117 | p=.000 | p=.000 | p=.222 | p=.000 | p= --- | p=.002 | p=.003 | | p=.747 | p=.299 | p=.389 | p=.262 | | p=.119 | p=.035 | p=.121 | p=.725 |  |
|  | Root:shoot | .4676 | -.4584 | .7197 | -.7338 | .2905 | -.8060 | -.6473 | 1.0000 | .6300 | | -.4587 | .2102 | -.2011 | -.3624 | | -.6413 | -.2869 | -.2374 | .2626 |  |
|  |  | N=21 | N=21 | N=21 | N=21 | N=21 | N=21 | N=21 | N=21 | N=8 | | N=8 | N=8 | N=8 | N=8 | | N=8 | N=8 | N=8 | N=8 |  |
|  |  | p=.033 | p=.037 | p=.000 | p=.000 | p=.201 | p=.000 | p=.002 | p= --- | p=.094 | | p=.253 | p=.617 | p=.633 | p=.378 | | p=.087 | p=.491 | p=.571 | p=.530 |  |
| Phenolic compounds | Salicin | .9070 | -.0254 | .8803 | -.7581 | .3368 | -.8555 | -.8927 | .6300 | 1.0000 | | -.3432 | .2150 | .1761 | -.9143 | | -.7886 | -.6901 | -.6618 | -.6073 |  |
|  |  | N=8 | N=8 | N=8 | N=8 | N=8 | N=8 | N=8 | N=8 | N=8 | | N=8 | N=8 | N=8 | N=6 | | N=6 | N=6 | N=6 | N=6 |  |
|  |  | p=.002 | p=.952 | p=.004 | p=.029 | p=.415 | p=.007 | p=.003 | p=.094 | p= --- | | p=.405 | p=.609 | p=.677 | p=.011 | | p=.062 | p=.129 | p=.152 | p=.201 |  |
|  | Rosm. acid | -.5313 | .6681 | -.5030 | .4618 | .1876 | .3537 | .1365 | -.4587 | -.3432 | | 1.0000 | .6759 | .8500 | .3790 | | .3058 | .6957 | .5407 | -.2209 |  |
|  |  | N=8 | N=8 | N=8 | N=8 | N=8 | N=8 | N=8 | N=8 | N=8 | | N=8 | N=8 | N=8 | N=6 | | N=6 | N=6 | N=6 | N=6 |  |
|  |  | p=.175 | p=.070 | p=.204 | p=.249 | p=.656 | p=.390 | p=.747 | p=.253 | p=.405 | | p= --- | p=.066 | p=.008 | p=.459 | | p=.556 | p=.125 | p=.268 | p=.674 |  |
|  | Esculin | .0319 | .4458 | -.0301 | -.1494 | .6212 | -.3653 | -.4209 | .2102 | .2150 | | .6759 | 1.0000 | .7622 | -.2265 | | -.4942 | -.0513 | -.2060 | -.5715 |  |
|  |  | N=8 | N=8 | N=8 | N=8 | N=8 | N=8 | N=8 | N=8 | N=8 | | N=8 | N=8 | N=8 | N=6 | | N=6 | N=6 | N=6 | N=6 |  |
|  |  | p=.940 | p=.268 | p=.944 | p=.724 | p=.100 | p=.374 | p=.299 | p=.617 | p=.609 | | p=.066 | p= --- | p=.028 | p=.666 | | p=.319 | p=.923 | p=.695 | p=.236 |  |
|  | Esculetin | -.0398 | .7466 | -.0600 | .0752 | .3908 | -.0638 | -.3541 | -.2011 | .1761 | | .8500 | .7622 | 1.0000 | -.1961 | | -.0870 | .3192 | .1601 | -.6350 |  |
|  |  | N=8 | N=8 | N=8 | N=8 | N=8 | N=8 | N=8 | N=8 | N=8 | | N=8 | N=8 | N=8 | N=6 | | N=6 | N=6 | N=6 | N=6 |  |
|  |  | p=.925 | p=.033 | p=.888 | p=.859 | p=.338 | p=.881 | p=.389 | p=.633 | p=.677 | | p=.008 | p=.028 | p= --- | p=.710 | | p=.870 | p=.537 | p=.762 | p=.176 |  |
| VOCs | 196 analyte | -.5999 | -.4498 | -.3256 | .4557 | -.3858 | .6283 | .4510 | -.3624 | -.9143 | | .3790 | -.2265 | -.1961 | 1.0000 | | .7899 | .4140 | .3284 | .4338 |  |
|  |  | N=8 | N=8 | N=8 | N=8 | N=8 | N=8 | N=8 | N=8 | N=6 | | N=6 | N=6 | N=6 | N=8 | | N=8 | N=8 | N=8 | N=8 |  |
|  |  | p=.116 | p=.263 | p=.431 | p=.256 | p=.345 | p=.095 | p=.262 | p=.378 | p=.011 | | p=.459 | p=.666 | p=.710 | p= --- | | p=.020 | p=.308 | p=.427 | p=.283 |  |
|  | 447 analyte | -.6085 | .0333 | -.5890 | .5565 | -.4814 | .8392 | .5961 | -.6413 | -.7886 | | .3058 | -.4942 | -.0870 | .7899 | | 1.0000 | .5379 | .3253 | .2437 |  |
|  |  | N=8 | N=8 | N=8 | N=8 | N=8 | N=8 | N=8 | N=8 | N=6 | | N=6 | N=6 | N=6 | N=8 | | N=8 | N=8 | N=8 | N=8 |  |
|  |  | p=.109 | p=.938 | p=.124 | p=.152 | p=.227 | p=.009 | p=.119 | p=.087 | p=.062 | | p=.556 | p=.319 | p=.870 | p=.020 | | p= --- | p=.169 | p=.432 | p=.561 |  |
|  | 630 analyte | -.7083 | .3588 | -.6235 | .4849 | -.4680 | .5508 | .7411 | -.2869 | -.6901 | | .6957 | -.0513 | .3192 | .4140 | | .5379 | 1.0000 | .8871 | .0202 |  |
|  |  | N=8 | N=8 | N=8 | N=8 | N=8 | N=8 | N=8 | N=8 | N=6 | | N=6 | N=6 | N=6 | N=8 | | N=8 | N=8 | N=8 | N=8 |  |
|  |  | p=.049 | p=.383 | p=.099 | p=.223 | p=.242 | p=.157 | p=.035 | p=.491 | p=.129 | | p=.125 | p=.923 | p=.537 | p=.308 | | p=.169 | p= --- | p=.003 | p=.962 |  |
|  | 764 analyte | -.6257 | .2080 | -.3711 | .4207 | -.6057 | .3998 | .5936 | -.2374 | -.6618 | | .5407 | -.2060 | .1601 | .3284 | | .3253 | .8871 | 1.0000 | .1865 |  |
|  |  | N=8 | N=8 | N=8 | N=8 | N=8 | N=8 | N=8 | N=8 | N=6 | | N=6 | N=6 | N=6 | N=8 | | N=8 | N=8 | N=8 | N=8 |  |
|  |  | p=.097 | p=.621 | p=.365 | p=.299 | p=.112 | p=.326 | p=.121 | p=.571 | p=.152 | | p=.268 | p=.695 | p=.762 | p=.427 | | p=.432 | p=.003 | p= --- | p=.658 |  |
|  | Total VOC | .0060 | -.4018 | .1120 | -.3720 | -.0746 | -.0919 | -.1490 | .2626 | -.6073 | | -.2209 | -.5715 | -.6350 | .4338 | | .2437 | .0202 | .1865 | 1.0000 |  |
|  |  | N=8 | N=8 | N=8 | N=8 | N=8 | N=8 | N=8 | N=8 | N=6 | | N=6 | N=6 | N=6 | N=8 | | N=8 | N=8 | N=8 | N=8 |  |
|  |  | p=.989 | p=.324 | p=.792 | p=.364 | p=.861 | p=.829 | p=.725 | p=.530 | p=.201 | | p=.674 | p=.236 | p=.176 | p=.283 | | p=.561 | p=.962 | p=.658 | p= --- |  |

Cells with P<0.05 are in dark grey, P<0.1 in light grey and P>0.1 in white.

#Since correlation matrix was calculated from population means, resulting correlation coefficients and P values differ from those presented in the main text of the manuscript.

Stem no 3M = stem no after three month; Height 5M = stem height after five month

196 analyte = (Z)-3-Hexen-1-yl acetate, 447 analyte = (Z)-3-Hexenyl isovalerate, 630 analyte = Caryophyllene (E), 764 analyte = δ-Cadinene
